# Editorial: Endometrial thickness as a risk factor for pregnancy complications

**DOI:** 10.3389/fendo.2023.1216952

**Published:** 2023-06-05

**Authors:** Barbara Lawrenz, Human M. Fatemi

**Affiliations:** IVF Department, ART Fertility Clinic, Abu Dhabi, United Arab Emirates

**Keywords:** endometrial thickness, pregnancy complications, fresh embryo transfer, preterm delivery, low birth weight, frozen embryo transfer

## Introduction

Crucial factors for the implantation and maintenance of pregnancy are a good quality, euploid embryo and a receptive endometrium. Endometrial receptivity is driven by the hours of progesterone exposure after sufficient exposure to estrogen. There is no non-invasive tool to detect endometrial receptivity, therefore endometrial thickness (EMT) often serves as a proxy for endometrial receptivity. However, there is an ongoing controversy on the impact of EMT on the outcome of ART (Assisted Reproductive Technology) treatments, and a consensus on a “thin endometrium” is lacking (1–3).

## The main points of individual contributions

This series includes five papers, evaluating the impact of endometrial thickness (EMT) on the pregnancy course, with some of them focusing on specific patient populations.

The study of Xu et al. evaluated retrospectively the impact of EMT on trigger day on clinical pregnancy (CPR), live birth (LBR), and miscarriage rate in > 40.000 cycles of IVF/ICSI/fresh ET cycles. The authors included a good responder population undergoing their first ET cycle with embryos at different developmental stages. Patients with an EMT below 5 mm were excluded from the analysis. EMT categorization was into 11 categories, each comprising just a 1 mm difference in EMT. This approach might be susceptible to inter-observer variability in the EMT measurement, and the groups of patients with EMT below 8 mm were small in sample size compared with the other groups, which could cause outcome bias. Their analysis concluded that CPR and LBR may achieve optimal level when EMT was ≥ 12 mm but some adverse pregnancy outcomes in patients with an EMT ≥15mm was observed, especially for CPR.

Du et al. studied the impact of EMT in patients with fresh single blastocyst transfers and singleton LBs on the incidence of low birth weight (LBW) and neonatal malformation. EMT was categorized into groups of thickness ≤ 7.5 mm, 7.6 to 12.0 mm, and >12.0 mm. While the neonatal malformation rate was comparable between the different EMT groups, the neonatal birthweight in the smallest EMT group (≤7.5 mm) was significantly lower than in the other groups. Using a similar cut-off value of EMT (< 8 mm), Liu et al. (4) also described an increased risk of delivering small for gestational age infants in patients with an EMT < 8.0 mm. Causative LBW in patients with a “thinner” endometrium might be a disturbance in the process of spiral arterial vascular remodeling and placental development.

Early pregnancy complications (ectopic pregnancy and early miscarriage) after FETs were retrospectively evaluated in the context of EMT on the ET day by Song et al. Different endometrial preparation protocols (natural cycle (NC) or Hormonal Replacement Treatment (HRT)) and different embryo developmental stages were included in the analysis, hence no adjustment was done for the endometrial preparation approach. This might pose a bias, as patients with thin lining underwent significantly more HRT protocols, especially as recently published data demonstrate an increased risk for miscarriage in the HRT approach (5).

The study of Tian et al., which seeks to evaluate the impact of EMT on the ongoing pregnancy rate (OPR) in the context of maternal age must be judged cautiously. When EMT was below 8 mm, estradiol dosage was increased in HRT cycles; hence, the criteria used to include these patients or not in the study is not clear; additionally, this approach obviously biases the results right from the study design. Furthermore, the primary outcome (OPR) is unclear as OPR was defined as a live fetus identified by ultrasound on day 12.

Caution is also necessary while reading the study of Huang et al., in which authors investigated the impact of EMT on obstetrical complications in women with polycystic ovary syndrome (PCOS) and concluded that in this group of patients, decreased EMT was independently associated with an increased risk of preterm birth (PTB), LBW, and SGA. For endometrial preparation, either HRT or a modified NC using letrozole and ovulation induction was applied. Even though the use of the endometrial preparation protocol was significantly different in the different EMT groups, the type of protocol was not considered in the adjusted analysis. This is problematic as an HRT approach has a higher risk for hypertensive disorders, placenta accreta, cesarean delivery, preterm birth, and LBW, as compared to NC FET cycles (6).

## Synthesis and conclusion

In summary, further studies are warranted to investigate the effect of EMT on the ART outcome beyond the achievement of pregnancy, and unified EMT thickness categories would simplify the interpretation of the data.

## Author contributions

BL: writing the editorial. HF: reviewing the editorial. All authors contributed to the article and approved the submitted version.
